# Target Protein for Xklp2 Functions as Coactivator of Androgen Receptor and Promotes the Proliferation of Prostate Carcinoma Cells

**DOI:** 10.1155/2022/6085948

**Published:** 2022-04-11

**Authors:** Baisheng Sun, Yin Long, Ling Xiao, Jiazhi Wang, Qian Yi, Dali Tong, Ke Li

**Affiliations:** ^1^Emergency Department, The Fifth Medical Center of the General Hospital of the Chinese People's Liberation Army, Beijing 100071, China; ^2^Department of Urology, Daping Hospital, Army Medical University, Chongqing 400042, China; ^3^Institute of Hematopoietic Stem Cell of PLA, Department of Hematopoietic Stem Cell Transplantation, The Fifth Medical Center of the General Hospital of the Chinese People' Liberation Army, Beijing 100071, China; ^4^Medical Service Department of 63650 Army Hospital of the Chinese People's Liberation Army, Urumqi 841700, China; ^5^Emergency Department, Chongqing Traditional Chinese Medicine Hospital, Chongqing 400021, China

## Abstract

The activation of the androgen receptor (AR) pathway is crucial in the progression of human prostate cancer. Results of the present study indicated that the target protein xenopus kinesin-like protein (TPX2) enhanced the transcription activation of AR and promoted the proliferation of LNCaP (ligand-dependent prostate carcinoma) cells. The protein-protein interaction between AR and TPX2 was investigated using coimmunoprecipitation assays. Results of the present study further demonstrated that TPX2 enhanced the transcription factor activation of AR and enhanced the expression levels of the downstream gene prostate-specific antigen (PSA). TPX2 did this by promoting the accumulation of AR in the nucleus and also promoting the recruitment of AR to the androgen response element, located in the promoter region of the PSA gene. Overexpression of TPX2 enhanced both the in vitro and in vivo proliferation of LNCaP cells. By revealing a novel role of TPX2 in the AR signaling pathway, the present study indicated that TPX2 may be an activator of AR and thus exhibits potential as a novel target for prostate carcinoma treatment.

## 1. Introduction

Androgen receptor (AR), a member of the nucleus receptor protein family (also known as nucleus receptor subfamily 3 group c member 4; NR3C4), plays a critical role in the transformation and proliferation of prostate carcinoma cells [1]. Similar to other members of the nucleus receptor superfamily, the transcription factor activation of AR is modulated by cofactors or coregulators, and the interaction between AR and its cofactors plays important roles in the transformation and maintenance of prostate carcinoma [2, 3]. Thus, the specific cofactors of AR and their subsequent roles in prostate carcinoma must be determined [4, 5]. The targeting protein for xenopus kinesin-like protein (TPX2) is well-established as a microtubule-interaction protein that regulates the maintenance of cell morphology. TPX2 contains a conserved motif that allows interaction with microtubules [6, 7]. Previously, the interaction between microtubules and TPX2 has been considered as the main mechanism underlying TPX2 in the promotion of cancer cell proliferation and division [8, 9]. Results of previous studies have further indicated that TPX2 may function as a potential target for prostate carcinoma treatment [10, 11]. Results obtained by Zou et al. revealed that overexpression of TPX2 is associated with the progression of prostate cancer and a poor prognosis in patients, whereas Pan et al. demonstrated that targeting TPX2 suppressed the proliferation of human prostate carcinoma cells [10, 11]. To the best of our knowledge, the present study was the first to reveal a novel mechanism of TPX2 in mediating the proliferation of prostate carcinoma cells. In the present study, the effects of TPX2 on the transcription factor activation of AR were investigated, and the proliferation of LNCaP cells, a well-established endocrine-dependent prostate carcinoma cell line, was examined using numerous experiments. Results of the present study further extended the current knowledge of the TPX2/AR pathway and uncovered the potential of TPX2 in the treatment of prostate cancer.

## 2. Material and Methods

### 2.1. Clinical Specimens, Cell Lines, and Agents

A total of 30 paired clinical specimens (paired prostate carcinoma and nontumor tissues) were obtained by our lab from May 2017 to November 2018 via daily surgical resection. Written informed consent was obtained from all patients. The collection and use of these clinical specimens were approved by the Ethics Committee of Daping Hospital, Army Medical University, and all experiments were carried out following the Helsinki Declaration. LNCaP cells (a common human endocrine-dependent prostate carcinoma cell line) were gifted by Dr Fan Feng from Beijing 302^nd^ Hospital [12]. The plasmids and lentivirus particles containing full-length TPX2 or AR or the small interfering (si)RNA of TPX2 were prepared by Vigene Corporation. The luciferase reporters, ARE-Luc (androgen response element luciferase reporter) or PSA-Luc, were gifted from Dr Fan Feng in the Beijing 302^nd^ hospital. The androgen (an agonist of AR), dihydrotestosterone (DHT; a common endogenous androgen; cat. no. S4757), was purchased from the Selleck Corporation [12]. DHT was dissolved in DMSO and diluted using the phenol red-free DMEM (Thermo Fisher Scientific, Inc.) and supplemented with 10% charcoal-stripped fetal bovine serum (FBS; Hyclone, Cytiva) for cell-based experiments [13, 14].

### 2.2. Dual-Luciferase and Reverse Transcription-Quantitative (RT-Q) PCR Assays

LNCaP cells were transfected with empty vectors, TPX2 or siRNA targeting TPX2, cotransfected with the aforementioned luciferase reporters (PSA-Luc or ARE-Luc), and the cells were harvested for 24 h following transfection, for the subsequent luciferase assays. Luciferase experiments were performed using a kit purchased from the Promega Corporation, following the instructions provided by the manufacturer and the protocols provided by Cui et al. and Gao et al. [4, 15] Moreover, the expression levels of PSA or TPX2 were examined using qPCR. The RNA samples from clinical specimens were extracted using a PARIS™ kit (Thermo Fisher Scientific, Inc.), and the RNA samples were reverse transcribed using Multiscribe™ Reverse Transcriptase (Thermo Fisher Scientific, Inc.) agent. qPCR was subsequently performed according to the protocol described in the referenced studies [16, 17]. GAPDH was used as the loading control, and the expression levels of PSA or AR were normalized to the levels of GAPDH mRNA. Primers used in the qPCR experiments were as follows: TPX2 forward, 5′-ACCTTGCCCTACTAAGATT-3′ and TPX2 reverse, 5′-AATGTGGCACAGGTTGAGC-3′; GAPDH forward, 5′-GCACCGTCAAGGTGAGAAC-3′ and GAPDH reverse, 5′-TGGTGAAGACGCCAGTGGA-3′; and PSA forward, 5′-GTGACG TGGATTGGTGCTG-3′ and PSA reverse, 5′-GAAGCTGTGGCTGACCTGAA-3′.

### 2.3. Cell Culture and Colony Formation Experiments

LNCaP cells were cultured in DMEM containing 10% FBS (Hyclone; Cytiva) at 37°C in 5% CO_2_. For the colony formation experiments, LNCaP cells were transfected with plasmids and harvested and seeded into the 6-well plates (Corning) at ~2,000 cells per well. After seeding the cells into the 6-well plates, cells were cultured in DMEM with 10% FBS. Cells in the plates were cultured for 3-4 weeks, and the colony formation assays were carried out following the methods described by Feng et al. [18]

### 2.4. Immunoprecipitation and Western Blot Analyses

LNCaP cells transfected with plasmids (FLAG, FLAG-AR, or FLAG-TPX2) or treated with the aforementioned agents were harvested for Western blot analysis [19]. The complex of FLAG-AR/TPX2 or FLAG-TPX2/AR was separated from the system and the FLAG-AR, FLAG-TPX2, and TPX2 or AR in the complex were examined using the corresponding antibodies (Abcam). The protein samples were prepared, and Western blot analyses were performed following a standard protocol. The expression levels of AR, PSA, PTX2, or GAPDH were examined using the corresponding antibodies (Abcam). GAPDH was used as the internal loading control.

### 2.5. Cellular Subfractionation Assay

LNCaP cells were transfected with plasmids and treated with the solvent control or 10 nmol/L DHT for 30 min and harvested for the cellular subfractionation experiments following the methods described in the previous publication [20]. The accumulation of AR or TPX2 in the nucleus or the cytoplasm was examined using the corresponding antibodies (Abcam). Lamin A was used as the control for the nucleus subfraction, whereas *β*-actin was used as the control for the cytoplasm subfraction. western blotting images were quantitatively analyzed using the ImageJ software (National Institutes of Health) [21].

### 2.6. Chromatin Immunoprecipitation (ChIP) Sequencing

ChIP analysis was performed following a protocol provided by the ChIP kit (Upstate Biotechnology, Inc.) or Yang et al. [22] Briefly, LNCaP cells were transfected with plasmids and treated with solvent control or 10 nmol/L DHT for 20 min. Subsequently, cells were harvested for ChIP experiments, and immunoprecipitation was performed using anti-AR or anti-TPX2 antibodies. RT-qPCR amplification was performed using DNA extracted from the immunoprecipitation and primers flanking the PSA promoter. The primers used to examine the recruitment of AR or PTX2 to AREI in the promoter region are as follows: ARE forward, 5′-CCTAGATGAAGTCTCCATG-3′ and reverse, 5′-AACCTTCATTCCCCAGGACT-3′.

### 2.7. In Vivo Tumor Model

LNCaP cells were transfected with plasmids and seeded into the subcutaneous position of male nude mice (age, 4-6 weeks) to form tumor models [23]. Tumor tissues were harvested 6-8 weeks after injections. Tumor volumes were measured by tumor length × tumor width × tumor width/2. Tumor weights were measured using a precision balance.

### 2.8. Statistical Analysis

Statistical analyses were performed using the Bonferroni correction with two-way ANOVA, using the SPSS statistical software (version 8.0; IBM Corp). *P* < 0.05 was considered to indicate a statistically significant difference.

## 3. Results

### 3.1. TPX2 Enhances the Transcription Factor Activation of AR

**Figure 1:**
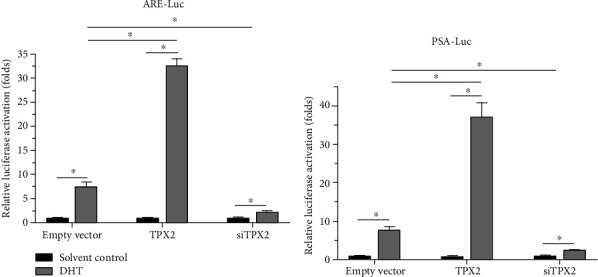
TPX2 enhanced the transcription factor activation of AR in LNCaP cells. LNCaP cells were cotransfected with empty vector, TPX2, or siTPX2 with luciferase reporters (ARE-Luc or PSA-Luc) and harvested for luciferase experiments. The activation of ARE-Luc (a) or PSA-Luc (b) was shown as mean ± SD. *P* < 0.05.

**Figure 2:**
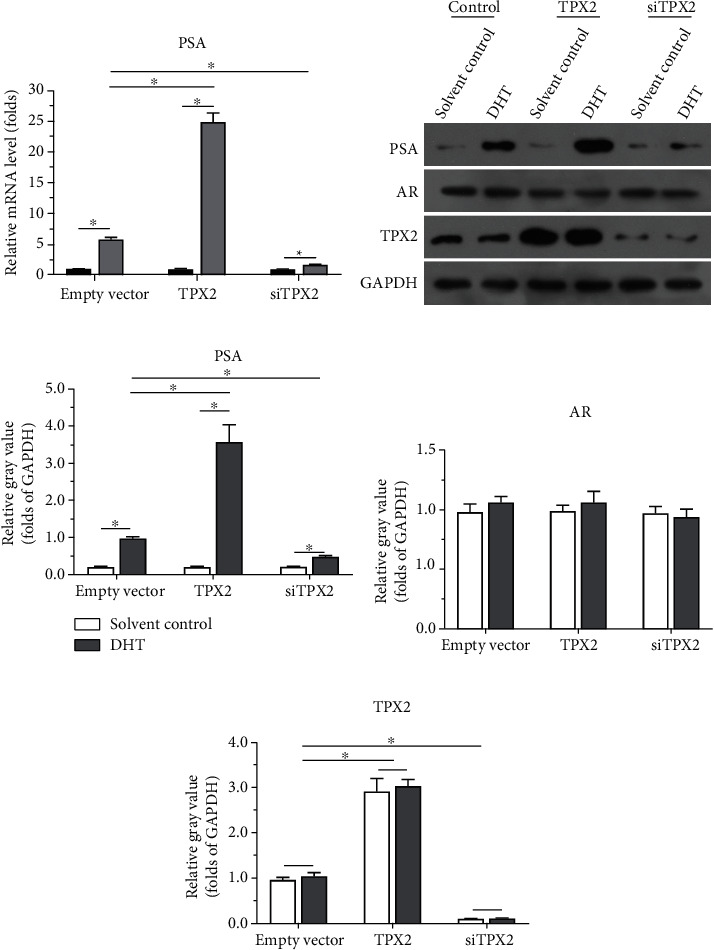
TPX2 enhanced the mRNA or protein expression of AR's down gene *PSA* in LNCaP cells. LNCaP cells were transfected with plasmids (control, TPX2, or siTPX2). Then, cells were harvested for qPXR or Western blot experiments. The mRNA level of PSA was examined by qPCR and shown as mean ± SD (a). The protein level of PSA or TPX2 was examined by Western blot, and the results were shown as the images of Western blot (b) or the quantitative results of the images (c–e). ^∗^*P* < 0.05.

The effects of TPX2 on the transcription factor activation of AR were examined. As displayed in Figure 1, overexpression of TPX2 enhanced the activation of luciferase reporters (ARE-Luc and PSA-Luc; Figures 1(a) and 1(b)) induced by DHT, a common endogenous androgen. Moreover, knockdown of TPX2 decreased the activation of luciferase reporters induced by DHT. Subsequent overexpression of TPX2 also enhanced both the mRNA and protein expression levels of PSA (Figure 2(a)), target gene of AR; however, knockdown of TPX2 also decreased both the mRNA and protein expression levels of PSA (Figures 2(b)–2(e)). Results are displayed as both Western blot analyses (Figure 2(b)) and quantitative results (Figures 2(c)–2(e)). Therefore, results of the present study indicated that TPX2 enhanced the transcription factor activation of AR.

### 3.2. TPX2 Interacts with AR

**Figure 3:**
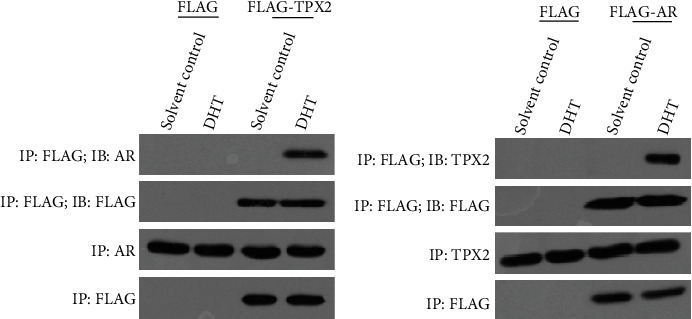
TPX2 interacted with AR in LNCaP cells. LNCaP cells were transfected with FLAG, FLAG-TOX2 (a) or FLAG, FLAG-AR (b) and harvested for the IP assays. The results were shown as images of Western blot.

Subsequently, the protein-interaction between AR and TPX2 in LNCaP cells was examined. As displayed in Figure 3, FLAG-TPX2 interacted with AR (Figure 3(a)), and the re-IP data demonstrated that FALG-AR also interacted with TPX2 in LNCaP cells (Figure 3(b)). Therefore, results of the present study demonstrated that TPX2 modulated the activation of AR via protein-interaction.

### 3.3. TPX2 Promotes the Accumulation of AR in the Nucleus and the Recruitment of AR to the Promoter Region of the Downstream Gene, PSA

**Figure 4:**
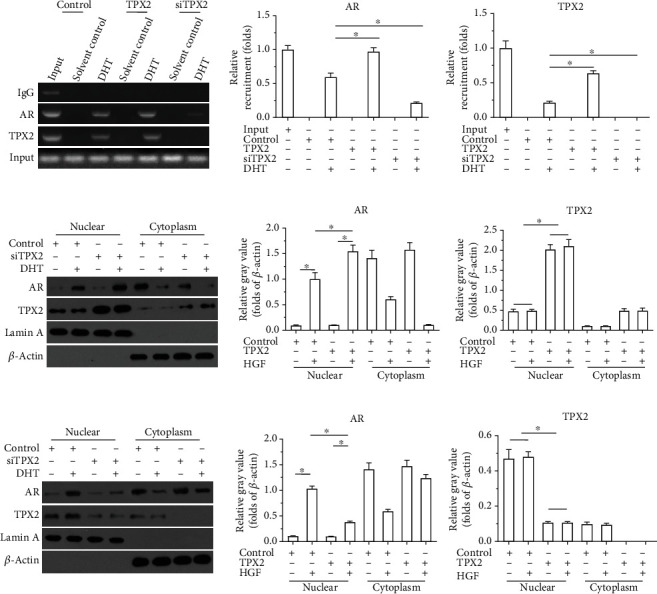
TPX2 enhanced the recruitment of AR to the PSA's promoter region or the accumulation of AR in nucleus. LNCaP cells were transfected with plasmids. The recruitment of AR to PSA's promoter was examined by ChIP (a). The accumulation of AR in nucleus was examined by the subcellular fraction (b and c). The results were shown as the images or the quantitative results. ^∗^*P* < 0.05.

To further examine the effects of TPX2, both subcellular subfractionation and ChIP assays were performed in the present study. As demonstrated in Figure 4, overexpression of TPX2 enhanced the recruitment of AR to the promoter region of PSA induced by DHT; however, knockdown of TPX2 decreased the recruitment of AR to the promoter region of PSA induced by DHT (Figure 4(a)). Moreover, the accumulation of AR in the nucleus was examined using a cellular subfractionation assay. As demonstrated in Figure 4, AR translocated from the cytoplasm to the nucleus when induced by DHT. Moreover, overexpression of TPX2 enhanced the translocation of AR from the cytoplasm to the nucleus induced by DHT (Figure 4(b)). In addition, knockdown of TPX2 decreased the translocation of AR from the cytoplasm to the nucleus induced by DHT (Figure 4(c)). Therefore, results of the present study demonstrated that TPX2 modulated the activation of AR by promoting the recruitment of AR to the promoter region of PSA or by promoting the accumulation of AR in the nucleus of LNCaP cells.

### 3.4. The Significance of TPX2

**Figure 5:**
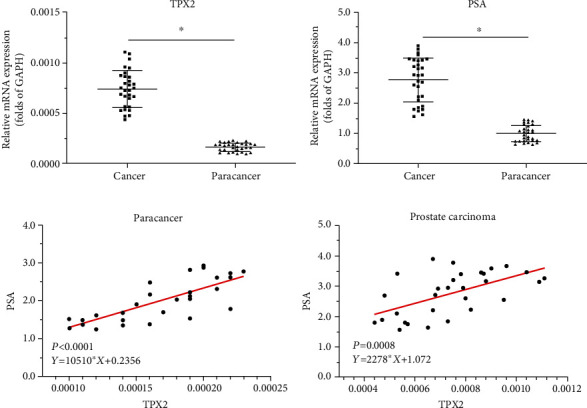
The clinical significance of the TPX2-AR axis. (a and b) The mRNA level of TPX2 (a) or PSA (b) in prostate carcinoma or nontumor specimens was examined by qPCR. (c and d) The relationship between the expression of TPX2 with PSA was shown as scatter plot images. ^∗^*P* < 0.05.

The aforementioned results indicated that TPX2 functions as a positive regulator (coactivator) of AR. To further verify the effects of TPX2, the expression levels of endogenous PSA or TPX2 were examined in clinical specimens. As displayed in Figures 5(a) and 5(b), the expression levels of PSA and TPX2 were increased in prostate carcinoma specimens compared with nontumor tissues. Moreover, the expression levels of TPX2 were positively associated with PSA both in cancerous tissues (*P* < 0.0001; *Y* = 10510∗X + 0.2356) and nontumor tissues (*P* = 0.0008; *Y* = 2278∗X + 1.072; Figures 5(c) and 5(d)). These results further confirmed the effects of TPX2 on AR.

### 3.5. TPX2 Increases the Proliferation of LNCaP Cells

**Figure 6:**
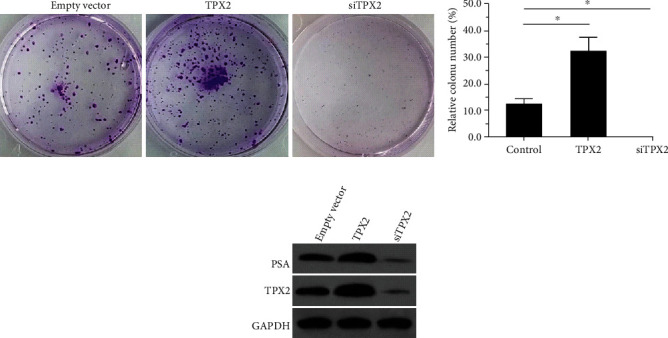
TPX2 enhanced the in vitro proliferation of LNCaP cells. LNCaP cells were transfected with plasmids and analyzed by the colony formation. The results were shown as the images of colonies (a) or the quantitative results of colony images (b). The expression level of PSA or AR in LNCaP cells was examined by Western blot (c). ^∗^*P* < 0.05.

**Figure 7:**
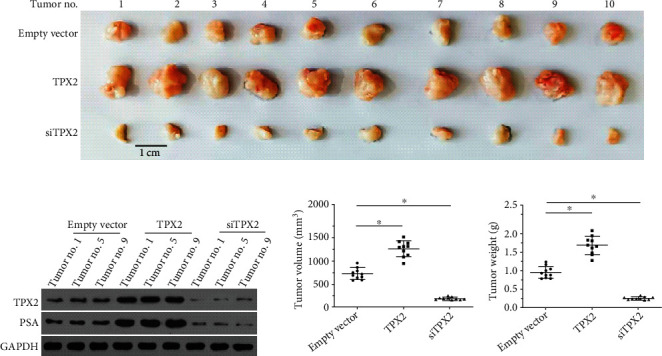
TPX2 enhanced the in vitro proliferation of LNCaP cells. LNCaP cells were transfected with plasmids and injected into the nude mice to form subcutaneous tumors. The results were shown as the images of tumor tissues (a), or the quantitative results of tumors according to the tumor volumes (c), or tumor weights (d). The expression level of PSA or AR in tumor tissues was examined by Western blot (b). ^∗^*P* < 0.05.

The proliferation of LNCaP cells was examined using both in vitro and in vivo experiments. As shown in Figure 6, overexpression of TPX2 enhanced the colony formation of LNCaP cells and enhanced the expression of PSA in LNCaP cells (Figures 6(a)–6(c)), whereas knockdown of TPX2 decreased the colony formation of LNCaP cells and the expression of PSA (Figures 6(a)–6(c)). Similar results were obtained using nude mice as a model. Notably, overexpression of TPX2 increased the subcutaneous growth of LNCaP cells and enhanced the expression of PSA in tumor tissues (Figures 7(a)–7(d)), whereas knockdown of TPX2 decreased the subcutaneous growth of LNCaP cells and the expression of PSA (Figures 7(a)–7(d)). These results are summarized as images of subcutaneous tumors (Figure 7(a)), Western blotting images (Figure 7(b)), tumor volumes (Figure 7(c)), and tumor weights (Figure 7(d)). Therefore, these results suggested that TPX2 increased the proliferation of LNCaP cells.

## 4. Discussion

**Figure 8:**
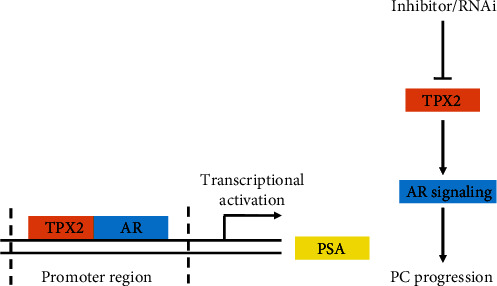
Schematic of TPX2 roles in prostate cancer progression. TPX2 enhanced the transcription factor activation of AR (a), inhibiting TPX2 is a potential therapeutic target against prostate cancer progression via AR signaling suppression (b).

Prostate carcinoma is one of the most malignant types of cancer and poses a great threat to male health [24]. As an endocrine-dependent malignancy, the AR pathway is necessary for the development and progression of prostate carcinoma, and the coactivators of AR have been characterized as key regulators of AR activation [25]. Therefore, discovering and elucidating the coactivators of AR is not only beneficial to clarify AR-related mechanisms but also enables more effective therapeutic strategies to be developed [25]. Results of the presents study indicated that TPX2 functioned as a newly identified activator of AR (Figure 8). TPX2 enhanced the transcription factor activity of AR in a ligand-dependent manner, whereas knockdown of TPX2 repressed the activity of the AR pathway. Moreover, overexpression of TPX2 promoted the proliferation of LNCaP cells. Therefore, the results of the present study indicated that TPX2 plays an important role in the regulation of the AR pathway activation and the proliferation of LNCaP cells.

It has previously been verified that the TPX2 gene, located at chromosome 20q11.2, is aberrantly expressed in several types of cancer, including prostate carcinoma [26]. Results of a previous study suggested that TPX2 enhanced the proliferation and division of cancerous cells by promoting the amplification of the centrosome or spindle apparatus (mitotic spindle) formation [27]. However, TPX2 could also function via other mechanisms. Results of further previous studies demonstrated that TPX2 enhanced the phosphorylation of AKT kinase (AKT) and increased the expression of alternative factors associated with the proliferation or metastasis of cancerous cells, including cyclin D1 or matrix metalloproteinases [28–30]. To the best of our knowledge, the results of the present study were the first to reveal a novel mechanism underlying the function of TPX2 in prostate carcinoma. Notably, TPX2 functioned as a coactivator of AR and promoted the translocation of AR from the cytoplasm to the nucleus. The cytoskeleton system is comprised of microtubules and is the basis of subcellular transport and localization of biological macromolecules in mammalian cells.

Numerous coregulators of AR have previously been identified, not only in regulating the activation of the AR pathway but also in regulating the proliferation of prostate carcinoma cells. These cofactors often function by directly modulating the transcription factor activation of AR, for example: (i) Modulating the interaction between AR and RNA polymerase II; (ii) modulating the interaction between AR and DNA; and (iii) modulating the widespread unfolding of chromosomes [31–35]. The translocation of AR in cells is of great significance to its well-established activity; however, very few studies have focused on identifying the specific coactivator that promotes the translocation of AR from the cytoplasm to the nucleus. Therefore, the present study extended the present knowledge of TPX2 and provided a novel theoretical basis for the mechanisms underlying the cofactors of AR.

Moreover, as displayed in Figure 4, TPX2 was examined both in the cytoplasm and nuclear subfraction of LNCaP cells. The results obtained from the co-IP experiment indicated that TPX2 could form a complex with AR (Figure 3). However, the specific conditions required for this complex and whether alternative proteins are involved require further investigation. Mass spectrometry will be utilized in future investigations to analyze these protein complexes [36–39]. Furthermore, TPX2 is considered to be closely associated with the function of microtubules, which are the basis for intracellular material transportation and subcellular component positioning [40–42]. Thus, future investigations should focus on determining whether the effects of TPX2 on AR are dependent on microtubules, and include drugs, such as paclitaxel or vincristine [43–45].

## 5. Conclusion

In conclusion, the present study revealed the interaction between TPX2 and AR in regulating the proliferation of prostate cancer cells. The results of the present study not only expand the current knowledge of TPX2 but also provide a novel theoretical basis for the development of prostate cancer treatments.

## Data Availability

The datasets used and/or analyzed during the current study are available from the corresponding author on reasonable request.
